# Forensic-Psychiatric Risk Evaluations: Perspectives of Forensic Psychiatric Experts and Older Incarcerated Persons From Switzerland

**DOI:** 10.3389/fpsyt.2021.643096

**Published:** 2021-06-14

**Authors:** Tenzin Wangmo, Helene Seaward, Felix Pageau, Lutz-Peter Hiersemenzel, Bernice S. Elger

**Affiliations:** ^1^Institute for Biomedical Ethics, University of Basel, Basel, Switzerland; ^2^Forensic Psychiatric Services, Solothurn, Switzerland

**Keywords:** older incarcerated persons, ethics, qualitative study, predictive risk assessment, forensic evaluation

## Abstract

**Background:** Forensic-psychiatric risk assessments of persons in prisons aim to provide treatment for their mental health disorders to prevent risk of recidivism. Based on the outcomes of such evaluations, it is decided, for instance, whether the person can be released or be assigned to further treatment with or without privileges. A negative evaluation would mean that the assessed person must remain in prison or in a forensic institution until his or her mental health has improved to live safely in the community. This paper highlights the process of forensic-psychiatric evaluations and the challenges faced by the two parties directly involved in this process in Switzerland.

**Methods:** Data for this manuscript are gathered using semi-structured one-to-one interviews. The study participants included a purposive sample of 41 older incarcerated persons under measures (i.e., mandated by court order to psychotherapeutic and psychiatric treatment), and 23 expert participants working in Swiss prisons or forensic institutions. We analyzed data using thematic analysis.

**Results:** Study findings within four themes are reported. First we describe the standards and procedures that expert participants use to carry out adequate risk assessments and conditions under which they refuse to perform such assessments. Thereafter, we present expert participants' concerns associated with predictive risk assessments and highlight the need to be cautious in drawing conclusion from them. We then reveal older incarcerated participants' reports on the inconsistencies with the forensic expertise and their belief that these reports tend to be negative toward them. The final theme concerns older participants' experiences of how these evaluations negatively impact their lives and their perspectives of a different future.

**Conclusion:** The study findings about forensic-psychiatric risk assessments point to the need for a clearer communication on how these evaluations take place and how decisions are taken based on them. As incarceration under measures denotes the necessity to continue therapy and reduce dangerousness, it is important that accused person understands his or her real progress, feel that the decisions are objective and justified, and are aware of the progress needed to achieve the goal of eventual release. Such clarity will not only be valuable for those under measures, but also the justice system.

## Introduction

Forensic-psychiatric risk assessment of persons in prisons, who are deemed dangerous due to their mental disorder and their crime, is ubiquitous (1). Structured instruments are often used to assist in examining risk and making risk management plans (2), but they are not without limitations (2–4). Forensic assessment informs judicial decisions made to incarcerate the person (i.e., sentence vs. measures, prolongation of measures), (further) treatment, privileges, and release from prisons (5, 6). The forensic mental health system is tasked with the role of balancing risk management to avoid future potential harm to others as well as providing treatment to incarcerated persons who suffer from severe mental health illness (3, 7).

Forensic-psychiatric risk evaluations lead to several ethical dilemmas. Horstead and Cree (8) discussed how patients in forensic risk assessments are passive actors where the assessments are done to them, affecting their progress. In such situations, patients believe that the purpose of risk-assessments is to further punish them, highlighting patients' opposite perspective (i.e., achieving freedom) as compared to that experts one (i.e., ensuring adequate evaluation). Another concern is that of dual loyalty: experts performing such assessments may in some rare cases also be the treating therapist (9, 10), or reports written by the therapists may be consulted in the evaluations. Treating therapists' dual loyalty dilemma in the Swiss context have been discussed in recent publications (11, 12). Also, studies have reported ethical concerns related to medical confidentiality and how professionals ensure clarity of their role as forensic-psychiatric experts (13–15). Since forensic psychiatric assessments are not error-free (16–18), it is important to preserve the rights of persons deprived of liberty to be freed when their punishments are served and their rights not to be unduly punished for crimes that they may or may not perpetrate in the future.

Brown and Singh (19) underlined three risk assessment approaches used in different contexts: unstructured clinical judgment, actuarial assessment, and structured judgment. Unstructured clinical judgment is an individual (and possibly subjective) evaluation of a client's likelihood of an adverse outcome without using any assessment tools. Here, clinical skills and experience are the key components. Actuarial assessment includes use of structured instruments that assess protective, risk, static, and/or dynamic factors associated with adverse events using statistical methods. Thus, this approach is objective and transparent, but often problematic because validation of the instruments has been done in a few countries (often the US) and thus can only partially be extrapolated to other contexts. Finally, structured professional judgment uses risk assessment tools to assess factors (risk, protective, static, dynamic) associated with adverse events (19). This allows to create scores which help experts to make categorical risk judgments (low, medium, high), but the latter are combined with clinical experience with the client. This is not as objective as actuarial methods, but less subjective - and therefore less biased - if compared to unstructured clinical judgement.

Upon comparing risk evaluations carried out by forensic experts in Switzerland using unstructured clinical judgment and psychology students using the Ontario Domestic Assault Risk Assessment tool, the latter group was more accurate in assessing long term recidivism among perpetrators of intimate partner violence than the experts (20). The findings point to the subjective nature of unstructured clinical judgement and how experts may not always be the best in judging an outcome. Several studies on risk assessment from Switzerland describe the validity and use of actuarial tools (such as PCL-R, HCR-20, Static-99, SORAG, VRAG) for the risk assessment process (21–25). A few studies describe the forensic-psychiatric evaluations and their shortcomings (4, 16, 17, 26), but they are not specific to Switzerland. There is no regulation defining which risk assessment tools need to be used in the country. Instead, forensic experts are free to decide the type of instrument that is most appropriate for a certain case. The overall assessment cannot be solely based on risk assessment tools, but needs to result in a differentiated individual case analysis (27).

We are not aware of any study that captures the perspectives of forensic-psychiatric experts and imprisoned persons in Switzerland on how forensic-psychiatric risk assessments are carried out and their consequences as experienced by those affected. Hence, our aim is to highlight the process of these forensic-psychiatric evaluations and the challenges faced by the two parties directly involved in this process: forensic-psychiatric experts and older imprisoned persons under so-called measures - a type of punishment present in the Swiss context that can theoretically be renewed several times and lead to a form of indefinite incarceration. Our objective is to provide nuanced understanding of the assessment process, how it is perceived and present ethical concerns that often remain hidden.

### The Context of Measures in Swiss Prisons

The Swiss Criminal Code (SCC) distinguishes penalties (imprisonment of a defined duration) from measures. Measures are applied if a penalty alone is insufficient to reduce the risk of recidivism and requires the offender to undergo treatment. Measures imply regular evaluations of the treatment's effect and of dangerousness. They are often regularly renewed and may thus result in a form of indefinite incarceration (when negatively evaluated and prolonged) of imprisoned persons to ensure safety of the general public.

Incarceration as a result of the application of a measure is possible under, for example, Article 59 SCC, which states that an in-patient therapeutic measure can be ordered, when its goal is to treat the underlying mental illness associated with the crime. This enables further incarceration until treatment has proven successful. Thus, upon successful treatment, the person can be released. When at the conclusion of a criminal trial it is determined that the mental health condition could not be improved with the treatments offered and/or if the person is considered very dangerous, a decision of indefinite incarceration may be pronounced (Art. 64 SCC). In case persons sentenced to indefinite incarceration are considered treatable at a later time, the decision can be converted to an in-patient therapeutic measure according to Art. 59. In such cases, s/he would receive mental health treatment, which theoretically opens the chances of a release in the future. Such treatment can be carried out in a forensic psychiatric institution, a penal institution, or a therapeutic measure center. Treatment will always be provided by mental health practitioners with a forensic specialization. The specific setting and interventions offered differ slightly between institutions, but all patients receive, at a minimum, individual therapy sessions. Some additionally receive milieu therapy and group therapy.

In the Swiss prison context, forensic-psychiatric assessments are carried out before imprisonment by forensic experts appointed by the court, in order to comply with requests from the court and its authorities. Forensic-psychiatric evaluation determine whether the measure should be prolonged or not and it occurs at least every 5 years. This evaluation is carried out by an external (forensic) psychiatrist and may include assessments by other professions such as forensic psychologists. These assessments influence decision-making at different levels (e.g., privileges, continuation of measures). According to Swiss law, forensic-psychiatric assessments require the expert to cover four aspects: (a) diagnosis of mental health disorder, (b) criminal responsibility and related link between psychiatric disorder and crime, (c) risk of recidivism, and (d) the necessity and the prospects of treatment success of the offender. Based on the expert's report on the above four points, a judge makes the decision on the specific case.

## Methods

The data included in this paper are part of the larger project “Agequake in Prisons - II” that aimed to capture the situation of older incarcerated persons serving security measures and those receiving mental health care in Swiss psychiatric and penal institutions. The project captured data from two participant groups: expert stakeholders involved in the provision of mental health care; and older persons receiving such care. An older incarcerated person is someone who is 50 years and older (28), and the emphasis on older persons is due to their rising number in prisons worldwide. However, as they still constitute a minority group in prisons, studies that seek their perspectives on the topic are lacking (29, 30). The overall project was approved as part of a multi-center study by the cantonal ethics commissions.

### Study Participants

The inclusion criteria for the older incarcerated participant group were: (1) 50 years and older; and (2) at least one contact with mental health service. These participants were recruited purposively from 11 of the 26 cantons (i.e., states) in Switzerland where data collection took place. From the included cantons, 15 prison and forensic institutions agreed to participate in the project and thereby support recruitment. We excluded correctional institutions that housed juvenile or remand prisoners exclusively as well as deportation centers. The older participants were contacted by members of the prison administration or the mental health service. These contact persons handed out the study information and informed consent to prospective participants. They also helped in scheduling interviews with older participants.

The expert participant group was also selected purposively and included those involved in mental health care provision in prisons and psychiatric institutions. The expert participant list was compiled using the Internet. All expert participants were first contacted *via* email or phone; thereafter, they received study information and the informed consent documents by email before the interview. Inclusion criteria were (1) background in mental health (psychiatry, psychology, or psychiatric nursing); and (2) substantial experience with incarcerated patients (minimum of 10 years work experience).

In total, 57 incarcerated older persons were interviewed and 31 interviews were carried out with expert participants. At the stage of data management, we excluded seven interviews from the older incarcerated group due to quality reasons, and two interviews from the expert group because they were not involved in mental health care of prisoners, but somatic care. At the stage of data analysis, out of the 50 interviews with older participants, data from 41 participants serving measures were used. Nine were excluded because those participants were sentenced to a penalty (and not a measure). They consequently had no experience with forensic-psychiatric evaluations. Similarly, we excluded the psychiatric nurses' transcripts, as they did not provide any information on risk assessment. Participant characteristics are presented in Table 1. A total of 68 experts were contacted representing a response rate of 45.6%. For the incarcerated older persons, we cannot provide a specific response rate because the research team only received information from our contact persons about older participants who agreed to participate and not about those who refused.

**Table 1:** Study participant characteristics.

Number of interviews	Incarcerated older participants	50 interviews • 14 Forensic institutions • 36 prisonsInterview length (in minutes) • M: 62 • Range: 16–120 • SD: 25.55 Period of data collection:December 2017–December 2018
	Expert participants	23 interviews • 6 Forensic institutions • 17 prisons Interview length (in minutes) • M: 72 • Range: 48–90 • SD: 14.16 Period of data collection:April 2017–January 2018
Participant characteristics	Incarcerated older participants	• 42 male, 8 female • Age range: 50–76 years; Average: 61 years • Sentencing: ° 41 serving security measures ° 9 custodial sentence
	Expert participants	• 19 male; 4 female • Professional training: ° 17 Psychiatrists ° 6 Psychologists
Language region	Incarcerated older participants	31 from German-speaking19 from French-speaking part
	Expert participants	16 from German-speaking7 from French-speaking part

### Data Collection Process

All interviews were conducted by two female research assistants (one of them HS), who were doctoral students at the time. They received qualitative interview training before starting data collection. Data collection was supervised by two main supervisors of the project, TW and BE, who have already led a similar project in Switzerland (31, 32).

The semi-structured interview guide was specifically developed for the purpose of the project, and was used by both interviewers to ensure consistency in the content and quality of data collection. The questions aimed at covering the various research objectives of the overall project. The interview guide was designed by two research assistants (one of them HS), approved by the senior researcher (TW), and pilot tested with three older participants and three experts. Afterwards, slight changes were made to phrasing or sequencing of the questions but no changes were necessary for the general content. Please see Table 2 for the topics covered during these interviews.

**Table 2:** Interview guide content.

**Incarcerated older persons**	**Expert participants**
• Typical activities and personal circumstances in prison and general conditions in prison circumstances. • Mental health care received, experiences with access to mental health care, level of care received, satisfaction with treatment, perceived stigma due to MH issues. • Perceived dual loyalty issues of the treating therapist. • Aging concerns in prisons: aging in prison including serving security measures and their regular experience with risk assessments, relationship with younger prisoners, opinions on work and free time activities offered, what are their future plans.	• Motivation to work in this context and work experience. • Information on organization of mental health care within the institutions they provide care, treatment characteristics, opinion on current access and provision of mental health care, influence of indefinite sentences on their health care provision, and patient motivation. • Experience and opinion on the provision of mental health care and forensic expertise for older incarcerated patients. • Role conflict associated with providing therapy as well as writing reports for the justice system. • Forensic-psychiatric evaluations.

Interviews with older incarcerated participants took place in the institutional settings and those with expert participants mostly in their offices. The language of the interviews was German, French or English, based on the preference of the participants. At the scheduled time of the interview, the researchers explained the purpose of the study, clarified that all data was treated confidentially, and that withdrawal was possible. Thereafter, written informed consent was obtained.

Interviewers and participants met for the first time on the day of the interview; there was no relationship prior to data collection. The research team offered no compensation to either group for their participation. However, incarcerated participants were paid the salary for missing the work hours by their institutions. All interview discussions with participants took place only once. There were thus no repeat interviews and interview transcriptions were not shared with the participants for further comments.

The number of interviews completed depended on the principle of data saturation (33, 34). We conducted data analysis alongside on-going data collection, during which we were able to identify that the new data being collected were not adding any further themes. Due to the larger national research project targeting multiple research questions (such as aging in the prison context and experiences with prison mental health services), we had to oversample in order to reach data saturation for the different topics covered. This resulted in data saturation with 50 interviews for the entire research project, which is a higher number of participants than one would expect. The sample included in the final analysis for this paper was 41 participants, representing an adequate sample size for qualitative research.

All interviews were tape-recorded upon consent. Project assistants transcribed these recordings into the language of the interview. Each of these transcriptions were checked for accuracy by another project assistant. All identifying information was removed during the transcription process.

### Data Analysis

For both sets of interview data, the analysis followed a similar process but separate coding trees were developed for each dataset. In the first stage of data analysis, five project team members (TW, HM, and 3 assistants) met to analyze eight interviews each from both datasets. This process allowed the team to ensure that there was a common understanding of the coding process and to develop a set of codes and memos. During this process, we discussed the different nuances that were visible in the data and sought to agree on how to name different codes, and what the codes meant - in case of complex code names. Thereafter, three study team members (SH/FP, TW, and HS) independently coded all the remaining transcripts and TW checked the completed coding tree to ensure consistency in the data coding process.

In light of the broad and explorative nature of the overall project (refer to Table 2, interview guide), a second level of further in-depth thematic analysis (35) took place for the topic of this paper.

Therefore, specific for this paper, the following analytical steps were taken. All data relevant for forensic-psychiatric evaluation coded in the first level were extracted. That is, we took out coded segments within themes such as “risk assessment/expertise” and “measures 59 and 64” from the expert dataset, and “management and legal issues” from the prisoner dataset. They formed the dataset for this manuscript on risk assessment. Although, the overarching theme names between the two coding trees differed, they were relevant for topic of risk assessment. TW reanalyzed all coded segments (i.e., the main theme and all sub-themes) and regrouped them into new themes and sub-themes. These were then discussed with the co-authors and further adapted to fit the scope of the manuscript. The final presented results are the outcome of careful discussions and agreements reached with all co-authors. We agreed on the following four themes relevant for the goal of this paper: (a) Process for an adequate forensic-psychiatric evaluation; (b) Risk of recidivism as the key expectation; (c) Concerns with the expert and the forensic-psychiatric expertise; and (d) Forensic-psychiatric evaluations - hopes crushed. To substantiate our findings, we present quotes from our participants. Longer quotes from participants are provided in the Tables, with one table corresponding to each theme.

## Results

### Process for an Adequate Forensic-Psychiatric Evaluation

All expert participants from both regions discussing the process of forensic-psychiatric evaluations stated that they use written sources of information to carry out their evaluations. The sources of information included medical/clinical reports and criminal files, that are normally made available to them. Furthermore, they participated in discussion(s) with the person being assessed. As part of their forensic-psychiatric expertise, some also complete question catalogs that contain different tools used to assess dangerousness to provide evidence for possible recidivism (Table 3, EPQ1).

**Table 3:** Participant quotes^*^ for theme “Process of forensic-psychiatric evaluations.”

EPQ1	Then uhm it is a matter of studying the files, in the sense of uh collecting information about the alleged offense and in addition gathering all further uh biographical or other information which are available to get a picture first of all. Then the next step would be to talk to the person under evaluation. At the first contact, consent would take place uh and then uh the expert evaluation happens […], then we take the entire anamnesis and also with regard to the alleged offense in order to answer the questions uh the list of questions [obtained from the justice system requesting the expertise]. And then with regard to the prognosis, there are of course various prognosis instruments that can be used depending on the alleged offense. (D18)
EPQ2	So, on the basis of the medical chart, the criminal file, and other information given by the physician, we have been able to do the expertise. […] But. There are situations where I don't really like to give my expertise without meeting the person. Even though, there is a judgment of the federal court which says that it is possible. … But in cases where there is not even a medical file, we only have the criminal file, which was made available by the justice system, there I tend to refuse, to say that, “no I won't (evaluate this person).” Because I would not do a good job, I would do a job that is not… I do not see on what … how I could make a reliable diagnosis like that. It's complicated. (F07)
EPQ3	P: Yeah. So I realize that if I rely on just one dossier, I have a tendency to be stricter when it comes to my risk assessment. Because I simply don't have the patient contact, I might not be able to give him the opportunity to show that he has changed, that he might react differently than what is written/portrayed in the files […]. (D24)
EPQ4	We never make an expertise alone - generally speaking. One of the two experts, he will be a bit in first line, if we can say it like that, who will meet the person under examination two, three, or four times. He will also read the file. And the other expert, he stays a bit in the second line and discusses the development with the first expert. He decides if there is need for other exams, if he is concerned with the progress of things. (F03)
EPQ5	Um, I discuss all my reports with colleagues. Anonymously, of course. But that can sometimes cause a bit of stomach ache, or, because it means, um, if a colleague of mine is on this case, he or she is already aware [of the patient]. [In such cases] would he disclose that he has already discussed it with me or not? So the forensic landscape is a small one and if you go for quality assurance, it also means that you are exchanging data and, among other things, you are a little [less] biased. But I do it anyway, because it's important to me (laughs) um, to really be able to exchange with colleagues. (D24)
EPQ6	Her [patient's] court case coming up soon, we will see if there will be a measure or maybe an outpatient measure. In her case, we [the forensic institution of the participant] was involved in both providing expert and treating professionals. This is not without problems, but uh in the end uh it was accepted like that. The forensic expert is one of my senior consultants from the outpatient sector. I supervised the expertise, but at the same time I am also responsible for the uh treatment, but uh not directly involved in the treatment. (D09)
EPQ7	So we get the uhm order [to carry out forensic expertise] from the public prosecutor's office or the court. uhm Then we check if we can accept it, if there are reasons for refusal or reasons why we can't do it, for example, if we know the person personally who is to be explored or examined or if we have treated him before. (D18)
EPQ8	Yeah, I mean creating a relationship should not be an illusion. The only relationship that we create with the person we assess, the person, s/he knows we are there to assess him/her. We know that we're here to assess him/her. So, it's not a therapeutic relationship either […] And then you also don't have to make the person believe that you are a nice doctor who is there to help him/her. Though afterwards you make a report saying that s/he is dangerous. So you need to set things straight from the beginning, that we are experts to answer to the judge's questions or the penal court. (F17)

* *EPQ, Expert Participant's Quote*.

One expert participant reported that s/he refuses to carry out an expertise if s/he has only access to the criminal file, but not to the patient or at least medical/clinical files. The reason for this was that an expert cannot come to a reasonable conclusion if all other sources of information are not available. In such cases (e.g., the refusal of the to be evaluated person to talk to the expert and the lack of a clinical evaluation in a medical record), this interviewee considers it inappropriate to come to any opinion on the diagnosis of possible mental health issue, a key component in their assessment, although, the law allows expert reports under such limited conditions. Another expert, who reported completing an expertise with only written information sources revealed that their final report is more conservative since it is based only on available documents and not a combination of written reports and discussions with the imprisoned person. Thus, the lack of opportunity to get an understanding of the incarcerated person with direct contact was viewed as a non-ideal way of making a forensic evaluation since it risks being harsher than normal. See Table 3 (EPQ2 and EPQ3).

In relation to the forensic-psychiatric evaluation procedure, expert participants from the French-speaking region revealed that two experts are involved in the evaluation. The goal is to ensure that the findings are as objective and unbiased as possible. In the German-speaking region, such evaluations are essentially carried out by one expert. Nevertheless, some participants noted having discussions with colleagues (keeping personal details of the prisoner confidential). These expert participants appreciated such informal discussions to ensure that their analysis of the situation is acceptable. See Table 3 (EPQ4 and EPQ5).

Although, expert participants reported that while court appointed experts during the trial cannot be treating therapists of the patient, it could be that the expert and the therapist are from the same institution. Such situations may occur when no other expert is available. However, a few expert participants explicitly stated that they refuse to complete an expert report for their own patients. In cases where the forensic psychiatrists carry out expert evaluations at the request of courts during trials, they noted that they ensure that the assessed person knows that there is no therapeutic relationship between them, and they are purely being assessed as agents of the justice system. See Table 3 (EPQ6–EPQ8).

### Risk of Recidivism as the Key Expectation

After the trial, once the measure is pronounced, according the interviewed experts the most important part of the report becomes the question of recidivism. One expert criticized that this is even the case during the trial. Although, therapeutic measures were conceived originally for those offenders that are not or only partially responsible for their acts and should thus not be punished but receive treatment, responsibility is neglected: “In the expertise, it is very clear, practically nobody is interested anymore in the question of responsibility. The only thing that people are interested in is the recidivism risk and the measures” (F03).

For many expert participants, highlighting the future dangerousness of a person was a difficult task. They equated it to forecasting and making “educated” guesses based on data that they know of from other studies. Participants underlined that such judgement of dangerousness and thereby the chances of recidivism is a subjective evaluation. Many clearly reported being very cautious in how they communicate this information. That is, they cannot and do not provide any quantified recommendation on recidivism and are only able to relay a sense of whether the risk is low, medium or high. Furthermore, several expert participants concluded that whether someone will recommit a crime depends on many factors such as the type of original crime, their mental health, how the patient works/worked with his own illness during the therapy, and individual factors (e.g., social network). Thus, the expert participants spoke about the conflict between how the justice system wants a precise prognosis of recidivism and their inability to do so. See Table 4 (EPQ1).

**Table 4:** Participants quotes^*^ for theme “Risk of recidivism as the key expectation.”

EPQ1	And then you have to look, what factors have played a role in the murder. If it was a relationship crime, does it have a connection with a personality structure, where an increased risk of violence is connected with it and so on. Did drug use also played a role? Well, there are a lot of factors that you have to take into account and then you think about, with regard to the individual, which are the favorable and which are the unfavorable factors. And the lawyers of course want an assessment that is as exact as possible and uh yes. From a psychiatric point of view, as exact as expected is usually not possible or is not allowed too, yes. (I: That's right, yes, yes) You can then, you can, you can, you just say it is low, it is moderate or it is high or it is very high. (D26)
EPQ2	No, well, in fact we use the instruments, only the instruments for which we have been trained specifically, so we have organized sessions, in fact specific trainings for a certain amount of instruments, those that are accepted, … on the aspects of resources, protective factors against risks, so we see that we have been trained for all those scales and then we use them in the framework of our expertise, but we use them in an extremely cautious way here at the center. On one hand because we have no validation points for these tools here in Switzerland, right, these are tools who have all the trouble in the world to be validated for prisoner populations in Switzerland. (F03)
EPQ3	Yes [I use risk instruments], but only to structure my decision, my assessment; so, SPJ, if that means anything to you, Structured Professional Judgement - a modern crime prognosis development has understood that in order to achieve the claim of individual assessment, it is of no avail if I, so to speak, only use the results of prognosis instruments, because then I do not take the individual case. I assign it to a group. That helps the authorities, but it does not help the court, which has to decide how to go forward with the case at hand. Um, and here it is useful to use the instruments, so to speak, to structure the decision-making process, not to overlook anything, to check all the relevant factors again. (D15)
OPQ1	Because we have a lot of “Mr. Little Gods” [“Herrgöttli”] here. The judges are fake and say “The psychiatrist says, there we do nothing else. For God's sake. I want to be re-elected. So let's bust him for something else.” (D43)
OPQ2	We call it the “small” measure (i.e., Art. 59, therapeutic measure). I know people who went in there five or six years ago, they never have any freedom, they never set foot outside, because they [the authorities, court] always say “yes this and this and that and” [are needed to be improved for final release]. […] I don't have to receive therapy in the sense of offense-oriented, two independent experts have confirmed that already. … And most of all, I want to know what comes at the end of the tunnel. Yes, not that you end up with “Yes, it's nothing, we're transferring you back to Article 64 [security measure],” in other words back to the prison system, I am not taking that risk and my nerves can't take this. (D54)
OPQ3	I'm hoping for this court date, that it goes well so far (coughs). The report of the, Mr. [name of expert] has made an expert opinion, … [stating] “A conditional release is possible.” And then instead of releasing me or something like that, they put me into measure 59 and there I am now nine years I did that. (D79)

* *EPQ, Expert Participant's Quote; OPQ, Older Participant's Quote*.

The demand for a clear recidivism prognosis is driven by the notion of public safety, which is said to be an omnipresent factor that guides the justice system's decision making process. Many expert participants wondered about the relationship between the different aspects and the actual effect of measures on recidivism: “did the person have a mental disorder at the time of the event? … is this person dangerous? So, is he likely to reoffend? … Can we reduce recidivism by applying measures?” (F17).

Some expert participants also stated that the questions related to risk assessment need to be interpreted carefully. The reason being that these questions from risk assessment tools may not be validated for persons incarcerated in Switzerland. At the same time, they were aware that these tools could potentially be interpreted differently based on who is reading the results, for what purpose, and the person's ability to understand the nuances of such tools. Therefore, they voiced that risk communication based on the results of these tools need to be done with much caution. A participant revealed that (s)he finds the tools useful to structure his/her report and not to come to a conclusion. See Table 4 (EPQ2 and EPQ3).

Different from most expert participants, only one expert participant reported that (s)he recommends prolongation of security measures only when it is necessary and serves a particular purpose: “Measures are extended if and only if it makes sense. [.] I have seen that patients were released, where we thought that this would certainly go wrong […]. Considering that the measure was useless, we had to end it (D15).” Because of this safety imperative, measures are often prolonged and may be perceived as similar to indefinite incarceration.

One older participant stated “Article 59 is a life sentence in disguise, […] an electric chair in disguise” (F85). Similarly, many incarcerated older participants reported that their sentence was prolonged based on the post-trial forensic-psychiatric evaluations and that there was very limited to no hope of being released. These participants thus highlighted repeatedly that it is very difficult to get out of a measure. A few older participants even said that they no longer require therapy, but they still remain in prison and are not released. Their measure has continued with the renewal of Art. 59. In stating so, they underscore the fact that measure (both therapeutic measure and indefinite sentence) practically takes away the real prospect of a release. See Table 4 (OPQ1–OPQ3).

### Concerns With the Experts and Their Expertise

As evident from the results presented above, older incarcerated participants perceived forensic-psychiatric expert reports and the decision that results from them as a hurdle to their freedom. The older participants felt that experts write their reports as if they know the future and spoke critically about the experts. They doubted expert's competence and were bothered by the fact that their future depended on a report. One participant stated that such predictions about the future should not be done by psychiatrists: “Because these are things that are predictive and the prediction should not concern psychiatry. Psychiatry should be based on facts, not predictions. You destroy a lot of people like that” (F57).

The older participants' doubts about expert's quality of work was highlighted with the point that different experts tend to provide different reports. One participant noted, “when I look at this report, the only thing that I realize is that everyone who comes to me and wants to judge me will come up with something new” (D55). Hence, several participants asked how they could trust anyone's evaluation if these evaluations of dangerousness vary based on who is evaluating them. This point further brings forth the lack of standardization across different experts as well as the inherent problem that these evaluations are difficult and subjective (also discussed by the expert participants) and the fact that the decisions are made by the justice system. See Table 5 (OPQ1).

**Table 5:** Quotes^*^ for theme “Concerns with the experts and their expertise.”

OPQ1	When she [the therapist] did it [an evaluation with a scale], she had a score of 8 for me, while the expert had a score of 18 [on the same scale]. So, it is a big difference. Then she [the therapist] filled a report but the commission did not take her report into account. To minimize things, these people [at the commission] called her, “just a therapist.” […] So here they are [at the commission] always minimizing things. They discard certain things. Then as time goes by the people at the commission take the things that are the most serious for them. As time goes by, they accumulate things on you. Then in the end you are not the person that was described anymore. You are described as someone else. (F57)
OPQ2	They [judges] base their decisions on the expertise, on the therapy reports and anyway in the last years anyway extreme [negative case examples], that is what counts, in principle. […] It's not about how the persons behaved in prison, for example, a matter of whether they did their work well or what so on, etcetera. So to speak [these issues are] irrelevant. […] They are very negative now […]. Because everyone protects his own field and because it is also about the responsibility, in case something would happen, that is. [.] and this is clearly losing your job. Well, I've already been told in no uncertain terms, the authorities told me: “Look, if you go out and something happens, (I: Mmh) there's a big risk that I'll lose my job.” (D48)
OPQ3	Uh, no. This is the procedure I owe to the 2011 incident [when a prison social therapist was murdered by a patient during outside therapy visits]. I told you already, that I was allowed to go out. And today, if you want to get out of here, which is the case since 2011, since 2012, you have to go through an enormous procedure to get the permission to out. (D54)
OPQ4	The expert had written a forensic report, you know, but that's called a file report (I: Yes yes), that means, [his report was based on files made available to him] from the time of the report to the time back uh when the first crimes took place. He just picked out certain things, didn't he? And that [is what he] did afterwards/so basically he just always/he just picks out everything negative, you know, from the files, right? And out of that, he wrote the forensic report. (D63)
OPQ5	I had bad luck there, I had a super good assessor [being sarcastic] who would have preferred to do nothing at all. And he pretty much put a crap together in the report [.]. And half of the things were not true, so they did not correspond to the facts. And partly I had the feeling that in the report, he has somehow copied things from another person, that did not concern me at all. (D75)
OPQ6	Well you see, I was talking to you about the female doctor [name of a third person]. Well, it's a person I have been seeing for 20 months now. So I saw her many times when I was in preventive detention. She helped criticize the expertise that was done on me, by saying that they were wrong. Though those critics were never retained. Experts never took these [critics] into account. They rejected them. […]. Obviously for him it is a gain to make the expertise. It is a good gain. About 20–30 thousand francs by assessment. He [the expert] won't complain, will he? (F57)
OPQ7	And the public prosecutor then applied to the higher court, that security measure should be applied, you know, and the request was dismissed, right? But now he sits in the expert commission, now there in November, or and demands the same thing again, you know. Demands security measure again, doesn't he, and since the referring authority has just jumped on it, right? So I mean, how can it be that the public prosecutor's office, which is dealing with my case uhm, can place themselves on a commission. I actually think it's weird. (D44)
OPQ8	So the result of the FOTRES, right? And in my case it was said I was actually/the report said that I was not treatable, um (.) Yes. Repeat offenders and so on. So it's really just the negative, right? And um (clears throat) in the first trial I was also sentenced for the article 64, so to say for indefinite security measure and then I said: “I cannot accept this.” […] So, uh, I was held in security measure and normally if somebody is held in security measure, he needs to get two expert opinion. I only had one. So there the judge should have already said: “Wait, excuse me. You want security measure and I only have one expert opinion. Where is the second opinion?” (D63)

* *OPQ, Older Participant's Quote*.

Furthermore, many older incarcerated participants felt that these forensic-psychiatric evaluations are written with considerable hesitation in light of the chances of recidivism and the presence of the public safety imperative. To some of them, this meant that negative reports were generally written that would justify extension of measures. They stated that neither experts nor judges wish to take responsibility for any future crimes that may happen. They also provided national examples of negative events that have occurred in the recent past when released prisoners have recommitted a crime, which led to reduced chances for everyone in the future. That is, negative event(s) colors everyone's perspectives and affects all persons who are in the process of being evaluated by forensic experts. This also means that the incarcerated persons receive fewer privileges, or rather, fewer chances to prove that they have improved and deserve more privileges and eventual release. See Table 5 (OPQ2 and OPQ3).

Several older participants stated that they find expert reports completely unfair and unacceptable. According to them, the reports are written in such a way that negative points are highlighted and positive points are hidden away, as well as additional unrelated information added. Along the same lines, two older participants stated that even if there is a positive evaluation, it is rejected. Hence, participants felt that the decisions are picked to come to the worst outcome for the incarcerated persons. See Table 5 (OPQ4 and OPQ5).

Other complained about procedural errors and reports being inadequate. They reported that decisions are taken behind their backs since the goal is to keep them imprisoned, thereby raising the issue of whether the entire expertise process is only a formality to confirm the intention to keep them in prison and not a truly open form of procedural justice. Older participants also questioned why experts would ever side with them since the experts are paid well for their work and that they have no interest in the incarcerated persons' future. See Table 5 (OPQ6–OPQ8).

### Forensic-Psychiatric Evaluations-Hopes Crushed

The forensic-psychiatric evaluations were viewed by the older participants as an ordeal, as prolongation of measures occurs every 5 years and in the absence of strong arguments advanced by the expert they continue to remain in prison. This thus results in the eventual building up of hopes and hopes being crushed each time. Some older participants described their situation as that of powerlessness. Despite good therapy progress and many years in prison, their much desired (and earned) release into society is weighed against public safety, where the latter ultimately triumphs since the perception of the justice system is that release is only possible if the risk of recidivism is zero. Therefore, forensic-psychiatric evaluation was a source of disappointment and frustrations. One participant concluded that it would have been better to hand out a life sentence of 40 years than to continuously renew a prison sentence using measures. See Table 6 (OPQ1–OPQ3).

**Table 6:** Quotes^*^ for theme “Forensic-psychiatric evaluations - hopes crushed.”

OPQ1	[…] S/he considers that there is a moderate/medium, high or other risk of recidivism. It's really decisive. I mean. There is no one who wants to go against the expert […] Listen it's not always easy to receive [the evaluation]. It is not always easy because the expertise doesn't necessarily go in the direction that you have wanted or wished for. Often it is the source of disappointment and frustration. (F82)
OPQ2	. measures Yes. (Breathes heavily) That's just. the curse of this custody. Isn't it? I/I have thought that back then I, well I still think the same today. So security measure is a torture, it would be smarter/I would have less trouble if the court said: “40 years imprisonment for the crime you have committed.” Right. But somehow like pfft, yes [you are] hanging in the air. (D78)
OPQ3	In the year 2000, there was again an Expert Commission meeting, and then they decided/As I told you I was still going a bit to a psychologist, as an alibi, or, and then they found “Yes, yes, we'll talk again in two years,” you know, because of a conditional release, right? And I actually assumed I would be released soon, because I could already go on vacation for one weekend every month and so I always met my son, started making plans there, or, and I actually assumed that I would be released in the next six months. Then there was this commission meeting “Yes, we'll talk again in two years,” or, then I thought, for what reason I was going to see the psychologist, you know. (D44)
OPQ4	That [not knowing] is, that is the biggest difficulty with the measure at the moment. (I: Mmh) That is the biggest, biggest difficulty right now. I have now like a time horizon just with eighteen months. In eighteen months I'm going to court again and then we'll see if the judge believes me or if he believes more, the authorities, who think I have an arson risk. After the many years in prison, I'm becoming as well more and more dangerous, [this is] the paradox. (D48)

* *OPQ, Older Participant's Quote*.

Finally, a few older incarcerated participants whose measures were renewed suggested that the decisions of the justice system were illogical. That is, even after their measures were prolonged a few times and although they believed they have made continued progress, their risk was not deemed to be diminishing. To them, the message of these judgements was that the more you stay in prison, the more dangerous you become (Table 6, OPQ4).

## Discussion

Unlike other studies that describe forensic-psychiatric risk assessments and their use for judicial decisions (6, 16, 17, 26), our findings provide empirical evidence on this evaluation process from two different participant groups involved in this process. This adds to the existing Swiss literature, which mostly focuses on the validity of risk evaluation tools (21–24). Overall, our results present important qualitative data to supplement knowledge from available quantitative studies questioning the value of predictive risk assessment (4, 17). At the same time, we identify several important gaps in the process of forensic-psychiatric risk assessment in Switzerland, and highlight critical concerns in this regard.

The judicial system has a difficult role to play in forensic-psychiatric evaluations. It is tasked to ensure the safety of the public, while assuring that the rights of the incarcerated persons are not violated and that they do not suffer undue harm (7) - described in some legislations as cruel and unusual punishment (36) or cruel, inhumane and degrading treatment (37). To ensure a fair process, objectivity is sought in these forensic-psychiatric evaluations. The goal of an expert's report is to provide critical information to then reach a legal decision. The role of forensic psychiatry in this evaluation process has been debated on ethical grounds (15, 38). In essence, these evaluations are used to form a judgement on, for example, whether incarcerated person shows improvements in his or her mental health condition that are sufficient to ensure public safety *via* reduced risks of recidivism. Our older participants complained that such risk assessments are no more than educated guesses and most experts admitted limits of their own evaluations. This is a concern that Stone (38) already raised against such evaluations more than three decades ago, and newer research underlines the lacking strength of such predictions (4, 39). Therefore, it is not surprising that the outcomes of this evaluation are said to be far from fair, with scholars questioning the weight placed on these evaluations by the justice system in deciding the fate of the person (18, 38). We note that expert participants cautioned against the use of risk assessment tools for coming to a decision as well, since averaged group values are not necessarily a reliable indicator of one individual person (17, 40).

Our expert participants were aware of the bias associated with interpretation of risk assessment tools and acknowledged that their forensic-psychiatric assessments suffered from several shortcomings (1, 3, 19). First, the procedure of carrying out this forensic-psychiatric evaluation lacked complete standardization. Most expert participants reported undertaking risk assessment when all sources of information were available in order to make the best judgment, a standard that is necessary to ensure fair process to the accused person. However, a few noted having to working with less information. These experts reported having to perform risk assessment with reduced information, such as lacking opportunities to meet the person under evaluation and/or unavailability of relevant files. As noted by one expert, evaluations tend to be harsher than usual when they are completed without meeting the person. The lack of access to the accused persons and/or their medical files is often due to their own refusal to allow such access to forensic-experts. It is unclear how to react in situations where the available material is too scarce to permit a professional judgement. Experts seem to use different standards regarding when to refuse the acceptance of an expert mandate. The risk that a few of them might agree to offer recommendations based on (too) limited material raises the ethical concerns of unfairness and undue further punishment.

Another concern with the post-trial forensic-psychiatric evaluations was that they were not always completed by third party experts with no relationship to the person under examination - thus potentially raising ethical dilemmas related to dual loyalty, which have already been discussed extensively in this context (9, 10, 41). Appelbaum (15) noted that it is acceptable for psychiatrists to perform forensic evaluation when there is no therapeutic relationship. This did not seem to be the case all the time as per the reports of our expert participants, but we cannot exclude that these might be exceptions. Also, since clinical reports written by therapists can be consulted in building the expert evaluation -irrespective of whether the expert is independent of the detained person or if they have a therapeutic relationship - the issue of dual loyalty may nevertheless remain, as the therapists' clinical judgment could potentially influence the legal outcome. We underline that in this paper, we did not include data related to dual loyalty, which is discussed elsewhere (11, 12).

To address the issue of subjectivity, some expert participants in the German-speaking region stated that they discuss cases with other experts. Although, this is a potentially beneficial process, the risk remains that the other expert may be able to identify the person under discussion, raising potential medical confidentiality concerns (13, 14). When the risk to confidentiality is evident in such situations, the question arises whether exposing the assessed person to such risk is justifiable, if this may help to reach a better outcome for the person under evaluation. In a similar but different manner, expert participants from the French-speaking region discussed how they conduct the evaluation in pairs to reduce subjectivity (19, 20).

Not surprisingly, subjectivity was also highlighted by the older incarcerated participants. They first stated that the reports differed based on the expert carrying out the evaluation, thus underlining subjectivity at the level of the individual expert. As a consequence, the future of the person under evaluation depends on a luck-factor associated with getting a “nice” evaluator. Inversely, if a person under evaluation receives a “difficult or overly cautious” evaluator, he or she may remain in prison for prolonged periods. This was noted by our older participants, who reported how difficult it is to get rid of measures. Such experiences of the imprisoned older participants concur with what Horstead and Cree (8) previously concluded: that incarcerated patients have lost faith in forensic risk assessment processes and believe that their aim is to further punish them. Moreover, these beliefs of the incarcerated persons that the system is working against them runs parallel to how the forensic reports are interpreted by the justice system, with an inherent presumption against the group under investigation (17).

The second level of subjectivity reported by the older participants pertained to experts “cherry picking” facts from different available reports on the assessed person, in order to appeal to the *public safety* and *zero-risk culture* arguments. Such tendencies may reflect a form of caution by expert evaluators' deriving from negative events (recidivist events such as rape and murder committed by another imprisoned person) that are often publicized in the media. The experts fear a public backlash and are also wary of the risk of losing their jobs in case their judgment turns out to have harmful consequences. Given that the process of post-trial forensic-psychiatric evaluation occurs every 5 years, it is natural that the evaluated person hopes for a better outcome each time. However, if the outcomes are perceived as dependent not only on their progress, but also on events unrelated to them, this might crash their hopes and lead them to lose any prospect of a different or a better future. That external factors beyond their control at times take precedence in how their cases are handled (e.g., one negative case resulting in further punishment for all prisoners) reveals a problematic aspect in the system and points toward a problem of collective punishment, which is inhumane according to international guidelines (42).

### Limitations

The study employed a qualitative methodology where forensic experts and older persons in prisons relayed their perceptions and experiences with the risk assessment process and its outcomes. The participants were chosen purposefully. In light of the research design, we do not claim our findings to be generalizable to all contexts, and they do not depict the experiences or perspectives of all forensic-experts in the country as well as other prisoner groups (e.g., younger prisoners). Our findings are nevertheless informative for others carrying research on the topic. Furthermore, we cannot exclude social desirability bias, that is, our participants - particularly the older persons subject to measures - may have forwarded the worst case picture to fit the general negative perception of the forensic-psychiatric risk evaluation process. To limit the influence of social desirability bias from incarcerated participants, we emphasized anonymity as well as our independence from prison and mental health care staff. For this reason, we did not collect demographic data on length of imprisonment, index offense, and psychiatric diagnosis since these are sensitive data that may give the impression that the researchers are there to judge them. At the same time, we interviewed older incarcerated person subject to measures, which indicates their generally long prison stays may have an impact on their views. Similarly, our expert participants may also have provided a more neutral account of the process in general and avoided the extreme cases that may ultimately raise more critical ethical concerns.

### Future Research

Our study results indicate that the influence of subjectivity on the results of psychiatric assessments is omnipresent. One way to increase objectivity is to deploy more systematically standardize procedures, particularly in countries like Switzerland that have no national guideline instructing forensic-psychiatric experts in their assessment procedures. Such guideline could, for instance, provide information on clear conditions (e.g., not being able to speak to the person in question) where it is appropriate to refuse a request for performing the duties of an expert mandate. Furthermore, procedures on how to create greater possibilities for peer consulting between experts could be elaborated, particularly targeting related confidentiality issues. Additional efforts should be put into consolidating data on risk assessment instruments for the Swiss context to create a list of recommended tools to be used in the different language regions of the country. Finally, the conceptual question on whether psychiatric risk assessments are of value to the individual case despite the influence of subjectivity needs to be critically debated to enhance their applicability within the criminal justice system and to make sure that they actually serve the overall objective of the criminal justice system.

## Conclusions

Our results question the overall quality and value of risk assessment due to its inability to reach a clear, consistent, and objective, hence valid prognosis. Experts themselves see their work as “educated guesses.” Indeed, the evaluated persons are exposed to concrete problems potentially resulting from an inappropriate predictive risk assessment - they are continuously incarcerated and their prospects as well as hopes for a better or different future are taken away with each negative evaluation. Also, the accused may feel punished based on a future that no one can accurately know. Expertise thus ought to be objective and standardized to achieve fairness.

These perceptions of the value of predictive risk assessment point to the need for clearer communication of how the forensic-psychiatric evaluations and their decisions are formed. There is also a need to clarify and justify these decisions to the accused person. We recognize that - in order to be clearer when delivering results of their evaluation- experts will have to conduct a more objective evaluation. Since incarceration under measures denotes the necessity to continue the therapy and reduce dangerousness, it is important that the persons understands his or her real progress, and feel that decisions are objective, justified, and fair. They also need to be aware of the progress that s/he must relay in order to achieve the goal of eventual release. Such clarity will not only be valuable for the person under measures, but also the justice system, as releasing individuals who have truly improved is both just and cost-effective.

## Data Availability Statement

The raw data supporting the conclusions of this article will be made available by the authors, without undue reservation.

## Ethics Statement

The studies involving human participants were reviewed and approved by Ethikkommission Nordwest- und Zentralschweiz. The participants provided their written informed consent to participate in this study.

## Author Contributions

BE and TW designed the project. HS contributed to data collection. HS, TW, and FP were engaged in data analysis of the project. Paper specific data analysis was carried out first by TW and validated by all co-authors. TW wrote the manuscript. L-PH supported data collection and carefully checked data interpretation, in light of his expertise in the field. HS, L-PH, FP, and BE read draft versions of the manuscript, provided critical and useful suggestions to improve the quality and precision of our data analysis, and thus the quality of the overall manuscript. All authors approved the final version submitted for publication and take responsibility of its content.

## Conflict of Interest

The authors declare that the research was conducted in the absence of any commercial or financial relationships that could be construed as a potential conflict of interest.
